# The Rho-Rock-Myosin Signaling Axis Determines Cell-Cell Integrity of Self-Renewing Pluripotent Stem Cells

**DOI:** 10.1371/journal.pone.0003001

**Published:** 2008-08-20

**Authors:** Nicole Harb, Trevor K. Archer, Noboru Sato

**Affiliations:** 1 Department of Biochemistry, University of California Riverside, Riverside, California, United States of America; 2 Laboratory of Molecular Carcinogenesis, National Institute of Environmental Health Sciences, Research Triangle Park, North Carolina, United States of America; Baylor College of Medicine, United States of America

## Abstract

**Background:**

Embryonic stem (ES) cells self-renew as coherent colonies in which cells maintain tight cell-cell contact. Although intercellular communications are essential to establish the basis of cell-specific identity, molecular mechanisms underlying intrinsic cell-cell interactions in ES cells at the signaling level remain underexplored.

**Methodology/Principal Findings:**

Here we show that endogenous Rho signaling is required for the maintenance of cell-cell contacts in ES cells. siRNA-mediated loss of function experiments demonstrated that Rock, a major effector kinase downstream of Rho, played a key role in the formation of cell-cell junctional assemblies through regulation of myosin II by controlling a myosin light chain phosphatase. Chemical engineering of this signaling axis by a Rock-specific inhibitor revealed that cell-cell adhesion was reversibly controllable and dispensable for self-renewal of mouse ES cells as confirmed by chimera assay. Furthermore, a novel culture system combining a single synthetic matrix, defined medium, and the Rock inhibitor fully warranted human ES cell self-renewal independent of animal-derived matrices, tight cell contacts, or fibroblastic niche-forming cells as determined by teratoma formation assay.

**Conclusions/Significance:**

These findings demonstrate an essential role of the Rho-Rock-Myosin signaling axis for the regulation of basic cell-cell communications in both mouse and human ES cells, and would contribute to advance in medically compatible xeno-free environments for human pluripotent stem cells.

## Introduction

During early embryogenesis, a single celled totipotent zygote gives rise to multiple identical blastomeres that further acquire tight cell-cell communications through compaction stage [1]. After this stage, cells are segregated to develop the pluripotent inner cell mass (ICM), from which mouse and human embryonic stem (ES) cells are derived [2]–[5], and surrounding trophectderm committed to form placenta after implantation [6], [7]. The ICM subsequently becomes pluripotent epiblast that further acquires epithelial junctional systems and polarized cell architectures soon after implantation [8], and has been reported to be able to give rise to new pluripotent cell lines [9]. Because ES cells mirror the pluripotent stem cell functions both *in vitro* and *in vivo* [10], [11], they can be used as model systems to understand the mechanisms underlying basic cell-cell interactions in early mammalian embryos that allow limited or no access for in depth studies especially in case of human [12], [13]. Although detailed analyses of cell adhesion states at ultrastructural and functional levels have been conducted [12]–[14], little is known about the signaling pathways that regulate essential cell-cell communication machineries in ES cells.

Recent advances in stem cell technologies include a novel approach to directly reprogram differentiated adult fibroblasts to derive pluripotent stem cells *in vitro* [15]–[18]. Intriguingly, despite the fundamental differences in the sources of cells and derivation methods, these induced pluripotent stem (iPS) cells emulate not only the differentiation capacities and gene expression patterns seen in ICM-derived ES cells but also the formation of colonies morphologically indistinguishable from those of undifferentiated ES cells [15], [17], [18]. This observation raises the intriguingly possibility of yet unexplored direct molecular links between cell-cell contact regulators and cells that specifically retain pluripotency. Investigation of such a mechanism may illuminate our understanding of how cells develop multicellular communication systems during early embryogenesis. This in turn may lead to the developing new technologies for engineering the basic growth nature of pluripotent stem cells.

Rho family GTPases are the intracellular signal processors that convert the signaling input into mechanical forces essential for the regulation of cell-cell adhesion, polarity, mitosis, and migration, functions conserved from primitive amoeba to humans [19]–[21]. The most studied Rho family members include Cdc42, Rac, and Rho. Biochemically they are characterized by the Rho GTPase domain cycling between the GTP-bound active form and the GDP-bound inactive form. As only the active form can signal to the downstream pathways, they are considered the molecular switch that spatiotemporally integrates actin cytoskeletons and molecular motors. Although the critical roles of the Rho family proteins for stem cell functions in adult stem cells have been extensively studied [22]–[24], their precise roles in pluripotent ES cells remain to be determined. Because among the Rho GTPase family members, the Rho function has been implicated in early embryogenesis [25], we sought to determine the potential role of Rho signaling in the regulation of cell-cell adhesion machineries and stem cell functions using pluripotent stem cells as a model system.

## Results

### Rho-Rock signaling is required for the maintenance of cell-cell integrity in mES cells

**Figure 1:**
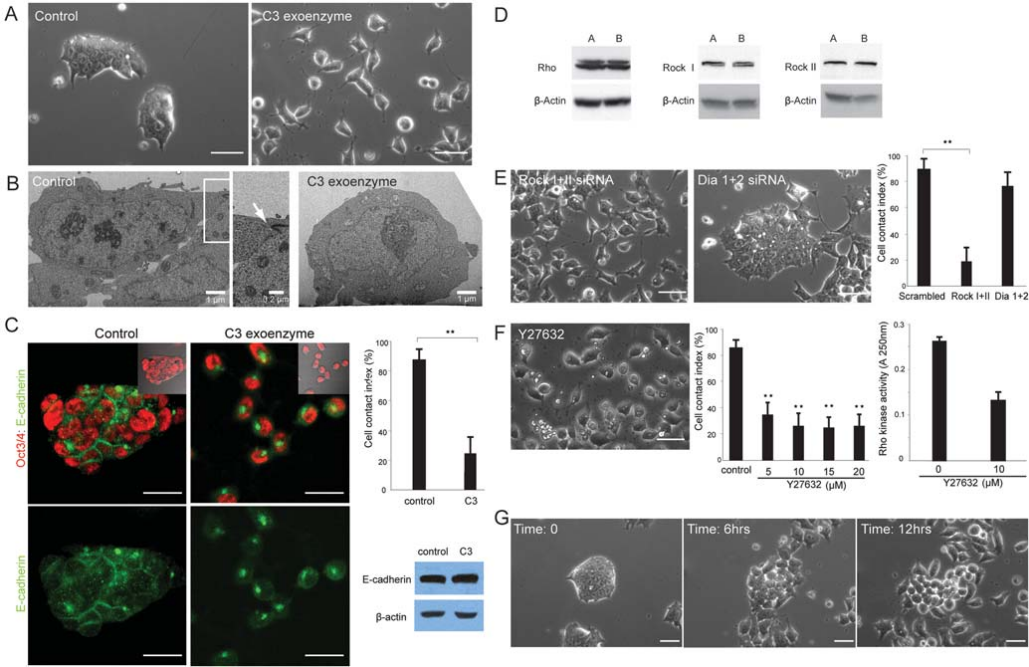
Inhibition of Rho or its downstream effector, Rock, leads to disintegrated cell-cell contact in mES cells. (A) Undifferentiated mES (CJ7) cells treated with control vehicle form typical round colonies with tightly associated cell-cell contact while treatment with C3 exoenzyme (20 µg/ml) for 24 hrs produced randomly distributed single cells with occasional physical cell-cell contacts. (B) Ultrastructure of undifferentiated mES cells in the colony analyzed by transmission electron microscope (TEM). Note that control cells are physically connected with large areas that include specialized cell-cell junctions (arrow). In contrast, a representative image of the C3-treated cells shows physical independency from neighboring cells. (C) Immunofluorescent images of undifferentiated mES cells. Oct3/4 and E-cadherin signals are demonstrated in red and green, respectively in top panels while E-cadherin (green) is solely shown in bottom panels. Note that E-cadherin is accumulated at cell-cell contact sites as seen in the intensified linear bands between adjacent Oct3/4-positive cells in the control condition whereas C3-treated cells mainly show cytoplasmic localization of E-cadherin. Cell contact index (CCI) represents the ratio of the number of Oct3/4-positive cells with linear accumulation of E-cadherin at their cell-cell contact sites to the number of all Oct3/4-positive cells. Data are mean±SD, **p<0.001, n = 5. The total protein level of E-cadherin remained unchanged in C3-treated mES cells as determined by Western analysis. β-actin was used as a loading control. (D) Rho, Rock I and Rock II were readily detectable in two independent mES cell lines (CJ7 [A]and E14 [B]) by immunoblotting analysis. β-actin was used as a loading control. (E) siRNA treatment targeting Rock I and Rock II in mES cells demonstrated marked disintegration of cell-cell contact reminiscent of C3-treated cells, whereas transfection of siRNA targeting Dia 1 and Dia 2 had little effects on cell-cell contact. Statistical significance was determined by comparing to the data of scrambled siRNA-treated cells. Data are mean±SD, **p<0.001, n = 5. (F) Morphology of mES cells treated with a Rock inhibitor, Y27632, at 10 µM for 24 hrs emulates the cell-cell disintegration seen in C3 or Rock siRNA-treated cells. CCI demonstrates the significant effect of Y27632 at low concentrations as compared to the control. Data are mean±SD, **p<0.001, n = 5. The effect of Y27632 on the kinase activity of Rock was determined by Rho kinase assay. Data are mean±SD, n = 3. (G) Time-lapse microscopic analysis shows the acquisition of cell motility at the expense of multicellular integrity over time. Scale bars, 25 µm.

To elucidate the role of the Rho signaling pathway, we specifically inhibited the endogenous Rho activity in ES cells by using Clostridium botulinum C3 exoenzyme which ADP-ribosylates and inactivates Rho without affecting other Rho GTPases such as Cdc42 and Rac [21]. Strikingly, mES cells treated with C3 exoenzyme demonstrated a remarkable decrease in cell-cell contact as compared with control cells and showed long tail-like cytoplasmic processes (Figure 1A). Similar results were obtained from three independent mES lines (data not shown). To clarify the state of cell-cell contact at a high resolution, ultrastructural analysis was conducted. Cells grown under the control condition established multilayered integrated cell assemblies with large areas of physical cell contacts. Cells especially in the outer layer of the colony were mutually interconnected by specialized junctional complexes (Figure 1B). In contrast, the majority of the cells treated with C3 exoenzyme were monolayered with no physical contact or occasional underdeveloped cell-cell contacts (Figure. 1B). To quantify the level of mature cell-cell contact in undifferentiated ES cells, subcellular localization of E-cadherin, a critical cell adhesion regulator in ES cells [12], and Oct3/4, a pluripotent-specific transcription factor [10], [11], [26], were detected by immunocytochemistry (Figure 1C). The representative images were evaluated based on the intensified linear accumulation of E-cadherin at the cell-cell contact site between adjacent Oct3/4-positive cells (cell contact index; CCI) (Figure 1C). The index was well correlated with the frequency of the formation of specialized junctional complexes between adjacent undifferentiated cells at the ultrastructural level (Figure S1). Consistent with phenotypic observations, the CCI was significantly decreased in cells treated with C3 exoenzyme while the total amount of E-cadherin protein remained unchanged (Figure 1C).

Rho regulates cell-cell adhesion through two major downstream effectors, Rho-associated kinase (Rock) and Diaphanous-related formin (Dia) [19], [27]. Rock and Dia were readily detectable by Western analysis or quantitative real-time PCR (QPCR) (Figures 1D and S2). In order to identify which pathway downstream of Rho is responsible for this cell phenotype, we used short interfering RNA (siRNA) technology. Robust transfection efficiency, a prerequisite to address the direct effect of siRNA at the individual cell level, was confirmed by using a control siRNA carrying green fluorophore (Figure S2). mES cells transfected with siRNAs targeting both Rock I and Rock II isoforms exhibited striking cell-cell disintegration comparable to that seen in C3 toxin-treated cells whereas cells transfected with both Dia1 and Dia2-targeting siRNA showed only weak effects on cell-cell contact (Figure 1E). The efficiency of each siRNA was verified by QPCR or Western analysis (Figure S2). These results indicate that in mES cells, Rock plays a major role in cell-cell adhesion downstream of Rho which contrasts with the essential role of Dia rather than Rock in non-pluripotent cell lines [27], [28]. In order to manipulate the Rock activity throughout the experimental period in a consistent manner, we decided to use a chemical compound, Y27632, which specifically blocks the Rock activity at low concentrations [29]. mES cells treated with Y27632 at different concentrations showed substantially decreased cell-cell integrity, which precisely emulated the phenotype seen in the cells treated with Rock-specific siRNA (Figure 1F), also verified by the ultrastructural analysis (data not shown). Similar effects were observed on testing with H1152, another Rock-specific inhibitor [29]. The reduction of the endogenous Rock activity in the compound-treated cells was determined by the Rho-kinase assay method (Figure 1F). In order to capture the dynamic transition of cell-cell contact states, time-lapse microscopic analysis was conducted. It was prevailed that the tightly integrated cells in colonies disassembled their intercellular connections while unlocking their concealed motile nature, suggesting that Rho-Rock signaling may also regulate cell motility besides physical cell-cell contacts (Figure 1G, S3A, and data not shown).

### Myosin II is the predominant effector downstream of Rock that mediates cell-cell adhesion

**Figure 2:**
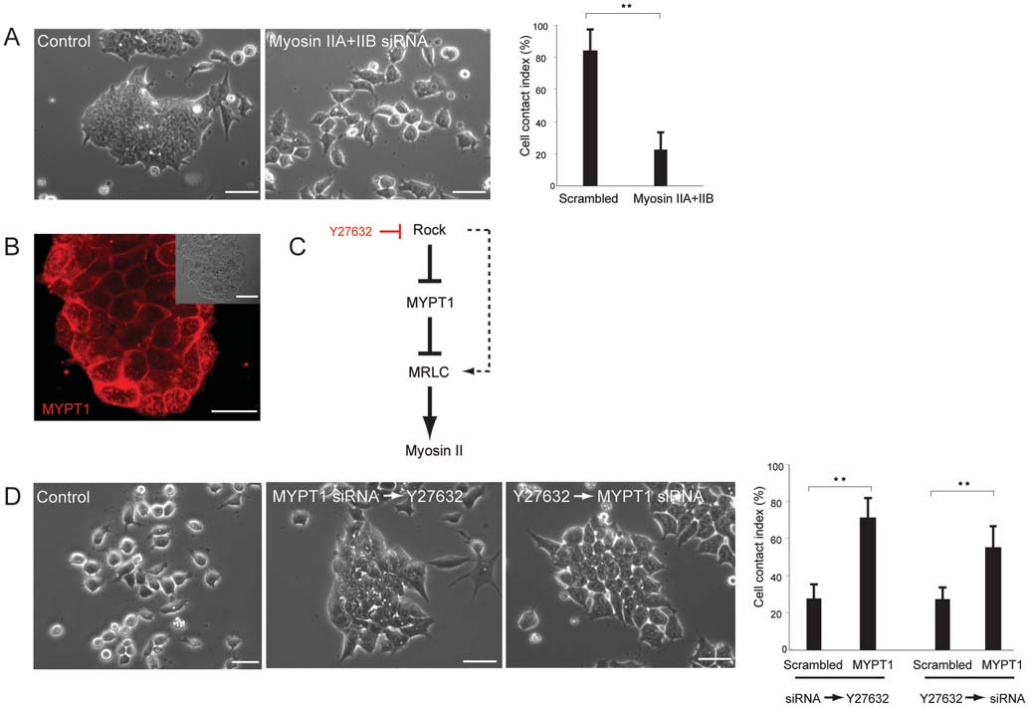
Myosin II is the canonical effector downstream of Rock in the regulation of cell-cell contact of ES cells. (A) Morphology of mES (CJ7) cells transfected with scrambled siRNA showing no effect on cell-cell adhesion whereas cells transfected with siRNA targeting both myosin IIA and IIB exhibited marked disruption of cell-cell contact. The cell contact index supports the morphological observations. Data are mean±SD, **p<0.001, n = 5. (B) Immunofluorescent analysis locates MYPT1 protein at cell-cell junction sites of undifferentiated ES cells. Inset shows the phase contrast image of the same colony. (C) A scheme depicting the molecular pathway by which Rock regulates myosin II function through the inhibition of MYPT1. The dotted line denotes the alternative Rock function that directly phosphorylates and activates MRLC. (D) In the protection experiment by siRNA, mES cells were transfected with MYPT1 siRNA, and 24 hrs later, they were treated with Y27632 for 24 hrs. The cells were able to maintain their cell-cell integrity against the strong cell-contact disruption effect of the inhibitor. In the rescue experiment, mES cells treated with Y27632 for 24 hrs were subsequently transfected with siRNA targeting MYPT1. Twenty-four hours later, cells were photographed. A substantial proportion of cells were able to restore their cell-cell contacts and formed pre-colony-like structures. The cell-cell contact states were quantified by CCI which represents the morphological observations. Data are mean±SD, **p<0.005, n = 5. Scale bars, 25 µm.

Cell-cell contact is directly regulated by various types of adhesion molecules and interconnected cytoskeletal machineries including myosin, ATP-driven molecular motors, which also determines cell architectures and polarized cell functions [30]–[32]. To understand the molecular mechanism by which Rock controls cell-cell contact in ES cells, we screened known downstream effectors of Rock [29], [33] by siRNA-mediated loss of function approach, and found the critical requirement of non-muscle myosin II for cell adhesion in ES cells. Myosin II, the two-headed conventional myosin, consists of three isoforms, IIA, IIB, and IIC, of which all, except IIC, are expressed in ES cells (Figure S2 and data not shown) whereas differentiated cells express all three isoforms [34]. Strikingly when myosin IIA and IIB isoforms were simultaneously depleted, cells showed remarkable disintegration of the cell-cell contact phenocopying that was seen in cells with loss of function of Rho or Rock (Figure 2A). In order to clarify that the myosin activity on cell adhesion is under the Rock regulation at the functional level, we focused on myosin phosphatase target subunit 1 (MYPT1), one of the major downstream targets of Rock that negatively regulates myosin function through dephosphorylation of myosin regulatory light chain (MRLC) [35]. Rock phosphorylates and inactivates MYPT1 to protect the phosphorylated, active form of MRLC that drives myosin II function. MYPT1 was found to be exclusively localized to cell-cell contact sites in mES cells (Figure 2B). If Rock controls the myosin function through inhibition of MYPT1 in mES cells, depletion of MYPT1 would rescue the intrinsic cell-cell communications from the robust effect of the Rock inhibitor (Figure 2C). Consistent with this hypothesis, depletion of MYPT1 before or after the inhibitor treatment resulted in significant protection or restoration of cell-cell contact, respectively (Figure 2D). Although a fraction of cells were insensitive to the siRNA treatment, it may reflect the alternative regulation of myosin II activity through Rock-mediated direct activation of MRLC [36].

### mES Cells can replicate independent of cell-cell contact while maintaining self-renewal ability when Rock signaling is repressed

**Figure 3:**
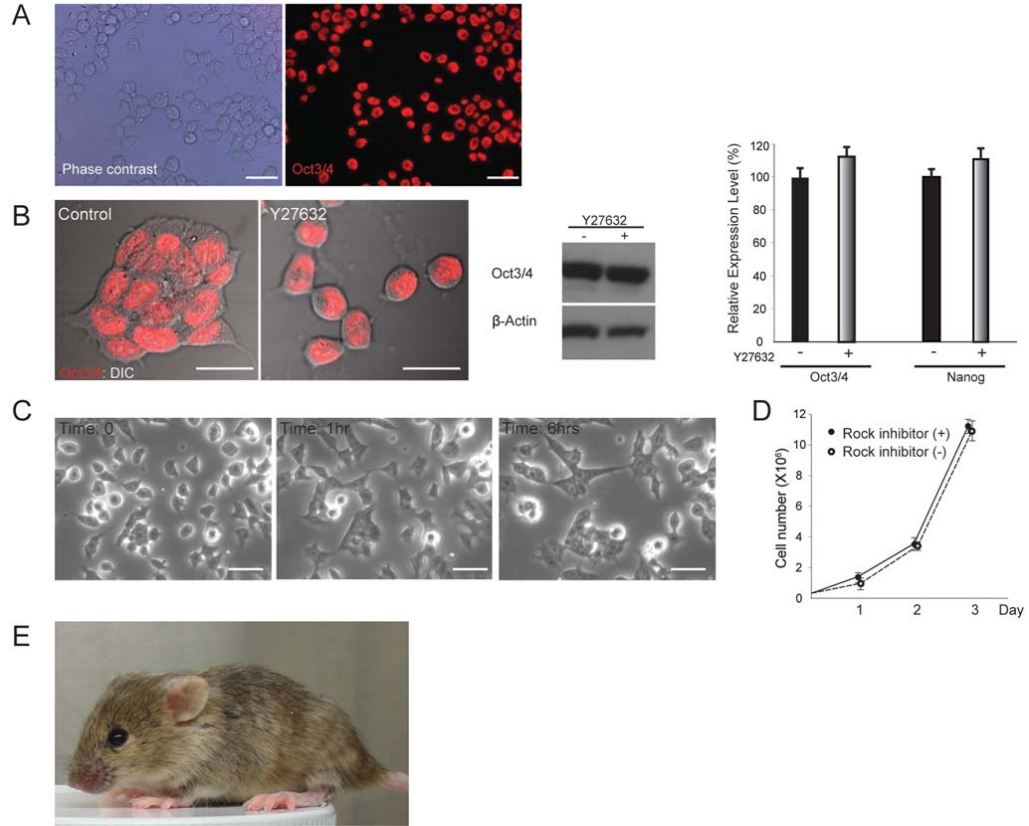
mES cells can self-renew without cell-cell contact by repressing Rock activity. (A) mES cells grown in the presence of Y27632 for 24 hrs. Each cell maintains the small round morphology and physical independency from neighboring cells. The immunofluorescent image demonstrates that virtually every single cell retains a strong Oct3/4 signal. (B) High-power confocal view of Oct3/4 immunostaining superimposed on DIC image demonstrates that mES cells passaged in the presence of Y27632 for 3 weeks maintain the Oct3/4 expression level equivalent to that of control, and high nuclear-cytoplasmic ratio. Findings are supported by Western analysis evaluating Oct3/4 protein levels between two conditions. β-actin was used as a loading control. QPCR analysis also confirmed the maintenance of the high levels of pluripotency-related gene expression in cells treated with Y27632. The expression levels (mean±SD, n = 3) are relative to the expression of genes in the control condition. (C) Sequential time images of mES cells. Cells were initially grown in the presence of Y27632. Subsequently, the inhibitor was removed from the medium (time: 0), and cells were monitored sequentially by the time-lapse microscope imaging system. Upon inhibitor removal, cells appear to search and capture each other to retrieve their direct intercellular communications in order to reestablish multicellular structures. (D) Growth curve of cells cultured in the presence of Y27632 is comparable to that of cells grown in the control condition. Each data point shows mean±SD, n = 3. (E) A representative chimera generated by injecting E14 cells grown in the presence of Y27632 for 3 weeks (7 passages) with occasional cryopreservation into C57/Bl6 mice-derived blastocyst. The dominant agouti coat color (E14 origin) indicates the strong chimerism. Scale bars, 25 µm.

Although mES cells treated with the Rock inhibitor greatly attenuated cell-cell cohesion, they retained a high nuclear-cytoplasmic ratio and compact cell morphology, which are common basic features of undifferentiated stem cells, suggesting that they still maintain pluripotent stem cell functions. Supporting this idea, despite their phenotypic changes, they retained strong expression of Oct3/4 in the individual cells (Figure 3A). To evaluate whether cells can undergo self-renewal under this condition, they were split at a low density and grown for several passages with occasional freezing and thawing. After multiple passages in the presence of LIF, cells still maintained the same morphology and strong expression levels of Oct3/4 and another pluripotency-specific transcription factor, Nanog [37], [38], whereas differentiation-related genes such as snail and N-cadherin involved in epithelial-mesenchymal transition were repressed (Figure 3B and data not shown). Importantly, when the inhibitor was removed from the culture medium, cells completely recovered their coherent cell-cell contact nature, which suggests the full reversibility of the effect of the inhibitor (Figure 3C and S3B). The dynamic transitional process after removal of the inhibitor was captured by the time-lapse microscopic analysis demonstrating that individually behaving cells gradually acquired multicellular constraints at the expense of independent motility somewhat analogous to the unicellular-multicellular conversion seen in the primitive social amoeba, dictyostelium [39] (Figure 3C and data not shown). Cells grown in the presence of Y27632 maintained a growth speed comparable to that of the control cells (Figure 3D).

The maintenance of pluripotency was determined *in vivo* by the chimera assay with mES cells that had been passaged in the presence of Y27632 at a low density for 3 weeks with occasional freezing and thawing. The microinjected cells were able to integrate into the host blastocyst and contributed to the generation of live chimeras (Figure 3E). Moreover, we confirmed that at least three male chimeras obtained from two independent microinjections were germ-line transmitters as determined by the agouti coat color of F1 offspring (Table S1).

Although coherent colony formation is the basic growth nature of self-renewing mES cells, these results have revealed that the cell-cell contact is dispensable for maintaining the pluripotent function when endogenous Rock activity is inhibited. In this regard, the previously reported cell contact-free growth of mES cells plated on E-cadherin fragment-coated dishes [40] may also account for the modulation of the Rock signaling because Rock and E-cadherin are known to mutually regulate to control cell adhesion machineries [41].

We have also evaluated whether alteration of Rock signaling influences self-renewal regulatory pathways in mES cells. Although in the Rock inhibitor-treated cells we found unaltered status of the positive self-renewal regulators such as LIF/Stat3 [10] and Akt [42], [43] as determined by the phosphorylation levels of the key signaling molecules, the activity of ERK signaling, a negative regulator of self-renewal in mES cells [10], was substantially attenuated, which suggests the potential involvement of Rock in differentiation machineries (Figure S4).

### Inhibition of Rock supports self-renewal of hES cells independent of animal-derived extracellular matrices

**Figure 4:**
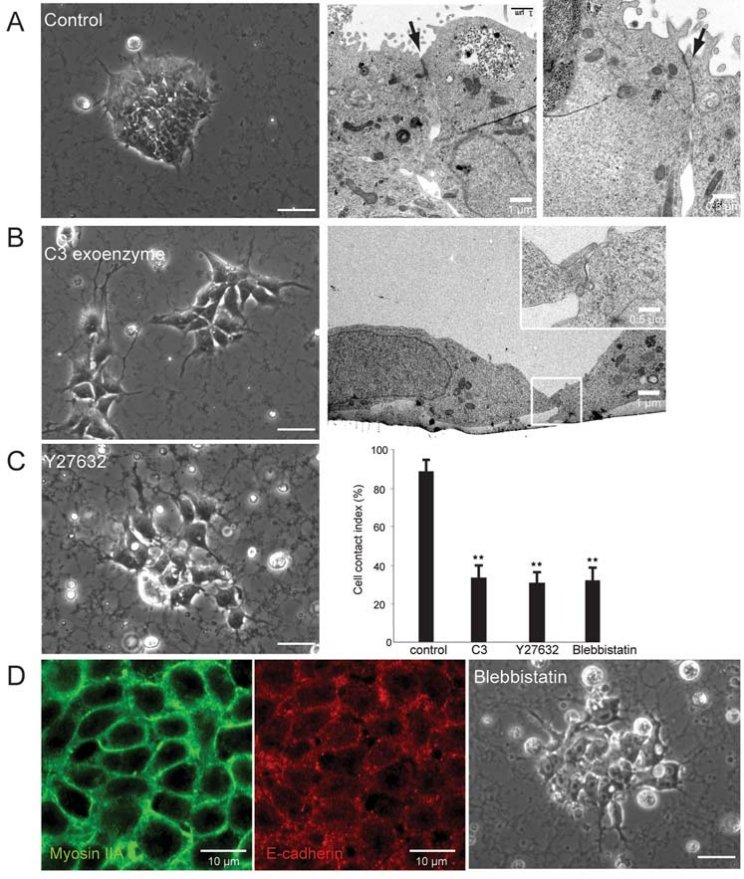
The Rho-Rock-Myosin signaling axis in the regulation of cell-cell communication is conserved in hES cells. (A) Morphology of undifferentiated hES cells (H9) showing a tightly connected colony. Ultrastructural analysis by transmission electron microscopy demonstrates specialized junctional complexes at cell-cell contact sites (arrow). (B) hES cells treated with C3 exoenzyme (20 µg/ml) for 24 hrs exhibited disruption of cell-cell connections. Ultrastructural analysis shows that C3-treated cells occasionally contact with neighboring cells through small areas at the cell periphery (inset). (C) Y27632 disassembled cell-cell junctions in hES cells grown on Matrigel at a higher (20 µM) concentration than that (10 µM) for mES cells. Cell contact index summarizes the effect of the inhibitors on hES cells. Data are mean±SD, **p<0.001, n = 5. (D) Immunofluorescence analysis of hES cells grown under the control condition show the exclusive localization of myosin IIA at cell-cell contact sites that overlaps with E-cadherin subcellular distribution. A myosin II-specific synthetic inhibitor, Blebbistatin (10 µM), disrupted cell-cell connections in hES cells similar to that seen in C3 or Y27632-treated cells. Scale bars, 25 µm.

Next we wanted to test whether the observed essential function of Rho-Rock signaling on cell-cell contact in mES cells is conserved in hES cells. To address this question, H1 cells grown under the feeder-free condition with Matrigel and conditioned medium (CM) derived from mouse embryonic fibroblasts (MEF) (Figure 4A) [44] were treated with C3 exoenzyme to eliminate the endogenous Rho activity. hES cells treated with C3 showed the clear cell-cell disintegration seen in mES cells, which suggests the conserved role of Rho signaling in human (Figure 4B). Rock-inhibitor treatment also resulted in the alteration of the cell-cell adhesion state although it required a higher concentration (20 µM) to promote visible phenotypic changes than that (10 µM) for mES cells (Figure 4C). Confocal analysis determined the colocalization of myosin IIA and myosin IIB with cell-cell borders in hES cells, which indicates their potential role in cell-cell communication (Figure 4D and data not shown). Consistent with this observation, treatment with a synthetic inhibitor specific for myosin II, Blebbistatin [45], led to remarkable cell-cell disintegration emulating the phenotype observed in the loss of function of Rho or Rock (Figure 4D).

**Figure 5:**
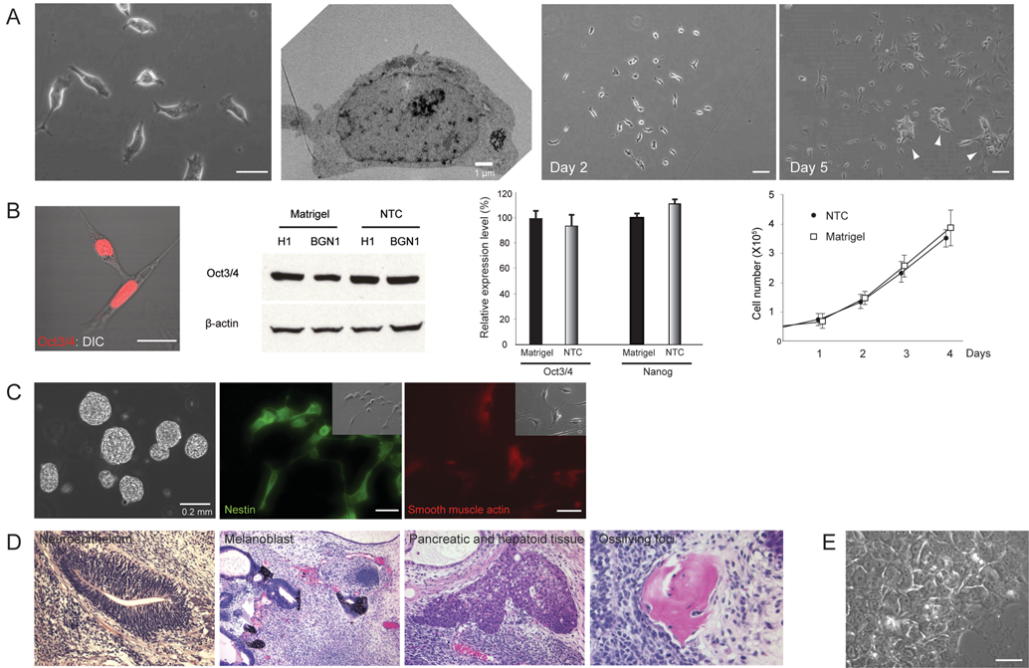
Self-renewal of hES cells requires neither cell-cell contact nor mixture of animal-derived extracellular matrices when endogenous Rock activity is inhibited. (A) Morphology of hES cells 3 days after plating on a non-tissue culture treated (NTC) plate without Matrigel-coating in the presence of conditioned medium supplemented with 10 µM of Y27632. Note that individual cells grow independent of physical cell-cell contact. Ultrastructural analysis shows that cells grown under this condition have a large nucleus encapsulated by a small volume of cytoplasm without cell-cell contact. Under this condition, hES cells initially grow in a separate manner (day 2), and subsequently, a fraction of cells form small clusters as the local cell density increases (day 5, arrow heads). (B) High power fluorescent confocal image demonstrates the maintenance of strong Oct3/4 expression in hES cells that have been passaged on NTC plates. Western analysis also confirmed the preserved Oct3/4 expression level in two independent hES cell lines grown on NTC or Matrigel-coated plates for several passages. β-actin was used as a loading control. mRNA expression levels of Oct3/4 and Nanog were evaluated by QPCR. The expression levels (mean±SD, n = 3) are demonstrated as relative to the expression of each gene in the control condition (Matrigel). The growth rate of hES cells grown on NTC plate at passage 32 for 4 days, or hES cells grown on Matrigel-coated plate for 4 days was measured by sequential cell number counting. The population doubling time was approximately 29–30 hrs for the cells grown on Matrigel and 31–33 hrs for the cells grown on NTC plate. Each data point shows mean±SD, n = 3. (C) hES cells grown in the presence of Y27632 for several passages were subjected to embryoid body formation assay without the inhibitor. Cells retained the ability to generate embryoid bodies in suspension culture, and to differentiate into neural (nestin-positive cells) and myogenic (smooth muscle actin-positive cells) lineages as determined by immunocytochemistry. (D) hES cells grown in the presence of Y27632 for 25 passages were subcutaneously injected into SCID beige mice. Six weeks after the injection, teratomas were harvested, and evaluated by histological analysis. Note that all three germ layer-derived tissues including neuroepithelium and aggregates of melanoblasts (ectoderm), focal islands of pancreatic and hepatoid tissue elements (endoderm), and ossifying foci (mesoderm), shown from left to right, were generated in the teratoma sample. (E) Morphology of hES cells passaged for multiple times on NTC plates in the presence of Y27632 and subsequently replated on Matrigel without the inhibitor. Cells restored intrinsic cell-cell communications and reassembled typical colony structures. Scale bars, 25 µm.

The observed requirement of a higher concentration of the Rock inhibitor needed in hES cells may be due to species differences or the different culture environments in which ES cells grow. For instance, Matrigel consists of a mixture of extracellular matrices (ECMs) [44] that may influence cell-cell or cell-substrate interaction forces. To test this possibility, we screened various culture substrates, and unexpectedly found that H1 cells grown on non-tissue culture treated (NTC) plates in CM with the Rock inhibitor were able to maintain individually separated cell growth at the same concentration as that used for mES cells while maintaining high nuclear-cytoplasmic ratio (Figure 5A and B). A similar effect was readily observed in BGN1 and H9 cells (data not shown). In contrast, when grown in CM without the inhibitor on NTC plates, most of the cells were unable to attach, or some adherent cells underwent spontaneous differentiation, which indicates that Rock may also involve the regulation of cell-substrate interaction [46] and differentiation in hES cells (data not shown). It should be noted that under this condition, while most hES cells maintained substantially less cell-cell contact, a certain fraction of the cells occasionally formed small clusters as the local cell density increased (Figure 5A) which differed from the discreetly separated growth of mES cells. This finding suggests that there may also be species differences in the regulation of cell-cell adhesion, which could be due in part to the differential involvement of Dia downstream of Rho, or to other Rho GTPases [21]. Their self-renewal abilities were validated by the expression of pluripotency–related molecules (Figure 5B), by the constant growth pattern over 30 passages with occasional cryopreservation (Figure 5B), and by multi-lineage differentiation capacities both *in vitro* by analysis of embryoid body formation (Figure 5C) and *in vivo* by teratoma assay demonstrating all three germ layer-derived tissues such as neuroepithelium and melanoblasts (ectoderm), focal islands of pancreatic and hepatoid tissue elements (endoderm), and ossifying foci (mesoderm) (Figure 5D). To test whether the effect of the inhibitor is reversible, hES cells grown in the presence of the inhibitor for several passages were replated on Matrigel in the absence of the inhibitor. Consistent with the reversible effect of the inhibitor on mES cells, hES cells completely restored their morphology (Figure 5E).

The observed tight cell contact-free growth on NTC plates may be advantageous to achieving spatiotemporally high-resolution analyses of the stem cell replication. To further explore the potential application of this method, we used a reporter hES line in which GFP is driven by Oct3/4 promoter. Through this system, the dynamic process of symmetric cell division as judged by equal inheritance of Oct3/4 transcriptional activity into two daughter cells was captured at a single-cell resolution independent of physically contacting neighboring cells (Figure S5). Because current self-renewal studies of ES cells largely rely on colony-based traditional culture methods that have limitations in dissecting cell-autonomous elemental functions due to the complexity of surrounding microenvironments [47]–[50], the new method in this study may provide unique strategies to decompose the essential functions of hES cells.

### Establishment of a defined environment for hES cells

**Figure 6:**
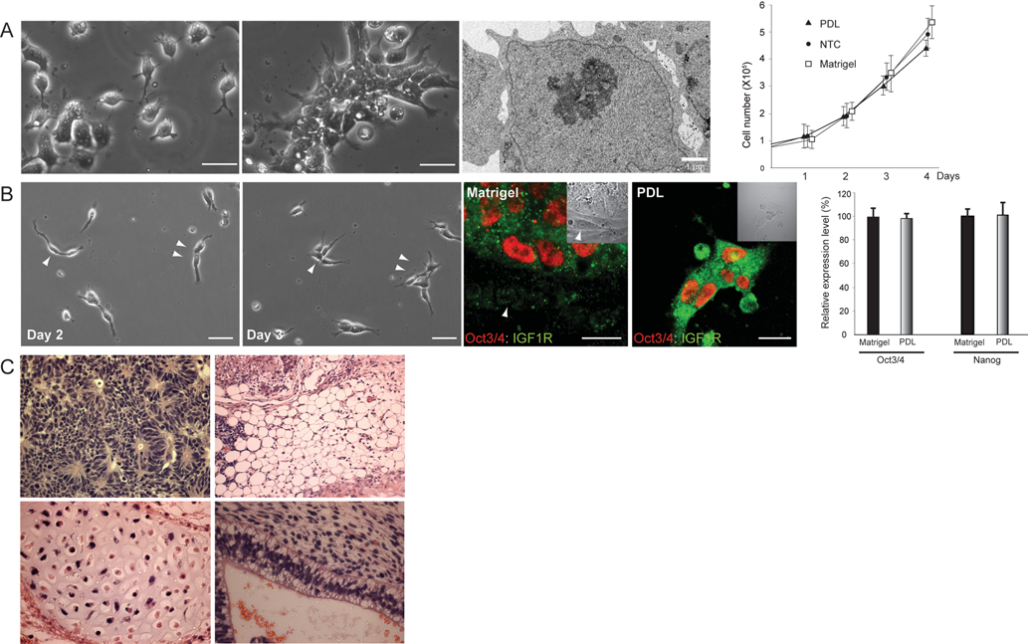
A culture method in which hES Cells self-renew in a completely defined condition. (A) Morphology of hES cells grown on a poly-D-lysin-coated (PDL) plate in a defined culture medium, mTeSR, supplemented with Y27632. Under this condition, cells initially grew without cell-cell contacts at a low density, whereas, within a few days, cells spontaneously reorganized cell-cell communications and formed small cell clusters with a greater tendency than that of cells grown on NTC plates. Ultrastructural analysis shows that hES cells grown on the PDL plate have underdeveloped cell-cell junctional structures. Cell growth curve indicates that hES cells cultured on PDL plates grow at a speed comparable to that of cells grown on NTC, and slightly slower than that of cells grown on Matrigel-coated plates within 4 days of the culture period. The population doubling time of cells grown on PDL plate was approximately 34.5–38 hrs. Each data point shows mean±SD, n = 3. (B) Sequential microscopic analysis demonstrates the replication of hES cells grown on a PDL plate in the absence of physically contacting fibroblastic differentiated cells. Arrowheads indicate the same cells in the different culture periods. The expression of IGF1 receptor (IGF1R) and Oct3/4 of cells grown on the PDL plate (PDL) was comparable to that of cells grown under the regular condition (Matrigel) as determined by immunocytochemistry. Oct3/4-negative fibroblastic differentiated cells (arrow) grown on Matrigel is negative for IGF1R. Insets show DIC images. QPCR analysis demonstrates that the expression levels of Oct3/4 and Nanog in cells grown under this condition (PDL) are equivalent to that of cells grown under the regular condition (Matrigel). The expression levels (mean±SD, n = 3) are denoted as relative to the expression of each gene in the control condition. (C) hES cells grown on PDL for multiple passages were subjected to teratoma formation assay followed by histological examination. All three germ layer-derivatives such as neuroepithelium (ectoderm), adipose tissue (mesoderm), cartilage (mesoderm), and gland-like epithelium (endoderm) are confirmed. Scale bars, 25 µm.

Although the culture method shown above can bypass the use of Matrigel, because it requires medium derived from MEFs, the method still relies on multiple undefined factors. To circumvent this problem, we examined whether the method using the Rock inhibitor could be combined with a reported completely defined medium, mTeSR which is identical to the total animal-free medium, TeSR, except for the use of bovine serum albumin [51]. Although cells were unable to adhere on NTC plates when grown in mTeSR probably due to insufficient scaffolding components, we found that poly-D-lysine coating efficiently supported the cell attachment and growth of BGN1 and H9 lines on the culture plate (Figure 6A). Under this condition, cells initially grew separately at a low density, and subsequently formed small clusters with a greater propensity than cells grown on NTC plates with CM (Figure 6A). Interestingly, throughout the extended period of passaging of two different hES cell lines, the vast majority of the undifferentiated cells replicated independent of close contact with Oct3/4-negative fibroblastic differentiated cells (Figure 6B). The maintenance of IGF1R, a major receptor for IGF II signaling required for hES cell growth [52], and strong pluripotency marker gene expression were determined by immunocytochemistry and QPCR, respectively (Figures 6B and S5). Thus, under this condition, hES cells may not require the proposed fibroblastic niche for self-renewal [52], but rather rely on their own autonomous regulatory systems as seen in mES cells. The *in vivo* multi-lineage differentiation capacity was confirmed after a series of passaging and cryopreservation by teratoma formation assay (Figure 6C). Considering that poly-D-lysine is a widely used synthetic polypeptide that is pathogen-free, inexpensive and easy to handle, as compared with the mixture of animal or human-derived ECMs, this new method may provide novel access to the practical animal-free environments critical for hES cell-mediated cell therapeutic strategies.

## Discussion

A tightly integrated colony formation has been considered one of the essential criteria to define the undifferentiated state of both mouse and human ES cells [4], [10]. Our findings have revealed that the Rho-Rock-Myosin signaling axis plays critical roles in the regulation of basic cell-cell interactions through which coherent colony structures are assembled. Moreover, *in vitro* and *in vivo* functional experiments have shown that self-renewal of ES cells does not require tight cell-cell communications in both mouse and human. It is of note that in non-pluripotent cell lines, Dia plays essential roles in the maintenance of cell-cell adhesion downstream of Rho whereas Rock oppositely disrupts the junctional complexes [27]. Given that another major Rho GTPase, Cdc42, also distinctively functions in ES cells as compared to their differentiated progenies [53], pluripotent stem cells in general may have unique signaling mechanisms regulated by Rho GTPases.

**Figure 7:**
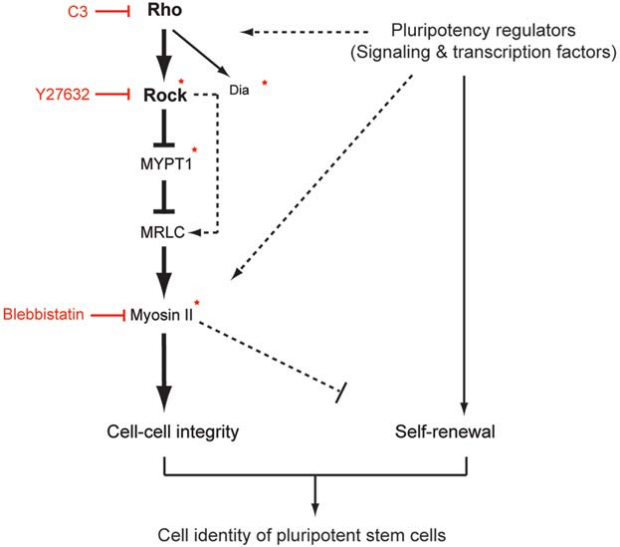
A model summarizing the Rho-Rock-Myosin signaling pathway that regulates basic cell-cell interactions in ES cells. Chemicals and siRNAs used in the study are highlighted in red and asterisks, respectively. Dotted lines indicate potential mechanistic interactions within or between the cell-integrity and self-renewal pathways, although further investigations are required to address these possibilities. Arrows denote activation and bars indicate inhibition.

To date several signaling pathways have been identified as the regulators for self-renewal in ES cells. As some of them are known to crosstalk with Rho or Rock signaling in mammalian cell lines [54]–[56], it is possible that the intrinsic cell-cell interactions in ES cells are also under the control of pluripotency regulators through modulating the Rho-Rock cascade. Intriguingly, activation of the Wnt/GSK-3 pathway by a synthetic compound, 6-bromoindirubin-3′-oxime (BIO) [57] substantially affected the cell-cell contact states in ES cells (unpublished observation) indicating the potential molecular link between self-renewal and intercellular communication systems in pluripotent stem cells at the signaling level (Figure 7).

We have determined that myosin II is the predominant effector molecule downstream of Rho-Rock signaling in the regulation of cell-cell contacts in ES cells. Whether the observed differentiation of hES cells grown on NTC plates without Y27632 also stems from the myosin II function remained to be investigated. Interestingly, our microarray analysis of hES cells showed substantial increase in myosin IIA gene expression when differentiated [58] (data not shown) suggesting the regulation of the myosin II function at the transcriptional level during differentiation. Consistent with this notion, mutant mice lacking myosin heavy chain IIA gene demonstrated embryonic lethality due to early differentiation and cell adhesion defects [59] further indicating the potential significance of myosin II in differentiation regulatory processes besides cell adhesion.

A recent study showed that the same Rock inhibitor we used has an anti-apoptotic effect on hES cells plated at low densities under the standard feeder-free condition or grown as cell aggregates in the suspension culture although the underlying mechanisms remained unaddressed [60]. The lack of the inhibition of cell adhesion by Y27632 in the previous study was probably due to the use of the regular feeder-free method relying on Matrigel-coating in which the applied concentration (10 µM) was not sufficient to affect cell-cell interactions, as was also seen in our experiment. We have, however, demonstrated that by combining NTC plates and CM, the same concentration of Y27632 was sufficient to impact cell-cell adhesion of self-renewing hES cells. Moreover, our demonstration of myosin II as the downstream effector of Rock may be also instrumental to the mechanism underlying the anti-apoptotic effect of Y27632 since myosin II has been implicated in apoptosis of several mammalian cell lines [61].

Finally, our findings also imply that the targeted manipulation of a specific signaling axis by a single synthetic compound can globally impact the basic cell-cell communication nature, and further evolve ES cells to reversibly self-renew in previously unadaptable novel growth environments. It is of interesting to test whether chemical engineering of the cell-contact regulatory mechanisms of ES cells developed in our study is also applicable to the recently established iPS cells or epiblast-derived stem cells [9]. Such a comparative analysis among different pluripotent stem cells in different species may contribute to understanding the fundamental mechanisms underlying acquisition of multicellular communication systems at the early stages of life and to further developing novel technologies that support new human pluripotent cell lines essential for therapeutic applications.

## Materials and Methods

### Chemicals and enzymes

C3 exoenzyme, Y27632, H1152 and Blebbistatin were purchased from Calbiochem (Gibbstown, NJ).

### ES cell culture

Three independent mES cell lines, CJ7 (provided by Willie Mark, Memorial Sloan-Kettering Cancer Center), E14 (provided by Tom Fielder, University of California, Irvine), and J1 (ATCC, Manassas, VA), were used for the experiments. mES cells were maintained on gelatin-coated dishes with the standard medium in the presence of leukemia inhibitory factor (LIF) at 1400 U/ml (Millipore Corporation, Billerica, MA) as described in detail elsewhere [62], or with ESGRO Complete Clonal Grade medium (Millipore Corporation) without further addition of LIF. Three independent hES cell lines, H1, H9 (WiCell, Madison, WI), and BGN01 (BresaGen, Athens, GA) were used for the experiments [57], [58]. A genetically modified hES line, BG01v/hOG (Invitrogen, Carlsbad, CA), that expresses GFP under the control of Oct3/4 promoter was also used. For the regular feeder-free culture, hES cells were grown on Matrigel (BD Biosciences, San Jose, CA)-coated plates in either the standard medium conditioned from mouse embryonic fibroblasts (CM) or a completely defined medium, mTeSR (StemCell Technologies, Vancouver, Canada) [51]. hES cells were regularly passaged by the standard method using dispase (Invitrogen) or trypsin-EDTA (Lonza, Allendale, NJ). The detailed protocols for hES cell culture under standard condition were published elsewhere [62]. For the microscopic, ulstrastructural, or immunofluorescent analysis, human or mouse ES cells were plated at approximately 5000–10000 cells/ cm^2^. When ES cells formed relatively small sizes of colonies, each of which consisted of approximately 30 to 100 cells, cells were subjected to chemical treatment or transfection experiments. For the animal-derived extracellular matrices-free culture, hES cells were plated at approximately 10000 cells/ cm^2^ on non-tissue culture-treated plates (BD Bioscience) with CM supplemented with Y27632 at 10 µM, or on poly-D-lysine-coated plates (BD Bioscience) with mTeSR supplemented with 10 µM of Y27632. Cells were passaged by using trypsin-EDTA in a relatively short period (3 to 4 days) to avoid the formation of small cell clusters. For the cell number count, cells were plated on 6-well plates in triplicates under the same condition as shown above. Cells were periodically harvested, stained with trypan blue, and counted by the hemocytometer.

### Immunocytochemistry

Cells grown on culture vessels were fixed in 4% paraformaldehyde (USB Corporation, Cleveland, OH). After washing with PBS/BSA, the samples were incubated with primary antibodies recognizing the target proteins at 4°C overnight. The primary antibodies used in the study include Oct3 (BD Biosciences), Nanog (abcam, Cambridge, MA), Rho A (Santa Cruz Biotechnology), E-cadherin (Millipore Corporation), myosin IIA (Covance, Cumberland, VA), myosin IIB (Covance), MYPT1 (Santa Cruz Technologies), Nestin (Millipore), Smooth muscle actin (Millipore), IGF1R (abcam). The samples were washed for three times, and incubated with appropriate secondary antibodies conjugated with Alexa Fluorophore (Invitrogen) at room temperature for 30 min. After three times washing, the samples were counterstained with 4′,6-diamidino-2-phenylindole (DAPI, Invitrogen), and examined by Nikon TE-2000-U fluorescent inverted microscope (Nikon Instruments, Melville, NY) equipped with CFI Fluor 40X objective or Zeiss LSM 510 confocal microscopy equipped with Apochromate water-immersion lenses (Carl Zeiss, Thornwood, NY). For the measurement of cell-contact index (CCI), E-cadherin and Oct3/4 localizations were visualized by a combination of each primary antibody and secondary antibody conjugated with Alexa Fluophore 488 and 555, respectively. The presence of intensified linear accumulation of E-cadherin at the cell-cell border of neighboring Oct3/4 positive cells was used as readout to assess the mature cell-cell junctions between the adjacent undifferentiated ES cells. The representative fluorescent images from each condition were initially taken by both confocal microscope and fluorescent inverted microscope to compare the consistency of the acquired images between the two different equipments. As their results were consistent in the evaluation of the above criteria, we decided to take advantage of the convenient operation of the conventional fluorescent microscope to take sufficient numbers of images required for the statistical analysis. Fluorescent images of approximately 50 to 100 cells in at least 5 different areas of each condition in three independent experiments were captured, and the total number of Oct3/4-positive cells and the number of Oct3/4-positive cells that met the above criteria were calculated in each image, and subjected to statistical analysis.

### Transmission electron microscopy

Mouse or human ES cells were grown on Thermanox plastic cover slips (Electron Microscopy Sciences, Hatfield, PA) that have been coated with gelatin, Matrigel, or poly-D-lysine (100 µg/ml, Millipore Corporation, Billerica, MA) as necessary. After rinsed with buffer (0.1 M Sorensen's phosphate buffer, pH 7.2–7.4), cells were fixed in 2% paraformaldehyde and 2.5% Glutaraldehyde for 1 hr. After washing, cells were post fixed with 1% OsO_4_ for 1 hr followed by dehydration in a series of graded ethanol. Cells were subjected to the infiltration for Spurr resin, and microsectioning. The samples were analyzed by Tecnai 12 transmission electron microscope (Philips, New York, NY).

### siRNA-mediated gene silencing

siRNAs specific for each target gene (siGENOME or ON-TARGETplus SMARTpool) were purchased from Dharmacon (Chicago, IL). The scrambled control or FAM-tagged siRNA (siGLO green transfection indicator, Dharmacon) was transfected into cells at a final concentration of 40 nM by using Lipofectamin 2000 and OptiMEM (Invitrogen) according to the manufacturer's protocol. Each siRNA targeting a single molecule was used at a concentration of 40 nM for each well of 6-well plates. When two distinct siRNAs were combined to deplete two different genes in the same well, each siRNA was reduced to 25 nM to keep the final concentration consistent throughout the experiment. The transfected cells were subjected to morphological or molecular analyses such as quantitative real-time PCR or Western analysis at 24 or 48 hrs after transfection. The sequences of siRNAs used in the study are shown in Supporting Information (Table S2).

### Quantitative real-time PCR (QPCR)

Total RNA was isolated from cells by using Qiashredder and RNAeasy mini kit (Qiagen, Valencia, CA). The extracted RNA sample was quantified by UV spectrophotometer, and qualified by the RNA Nano Lab chip (Agilent Technologies, Palo Alto, CA).

Two µg of total RNA was reverse-transcribed using SuperScript III RT-PCR system (Invitrogen) according to the manufacturer's protocol. Each cDNA sample in triplicates was PCR amplified with specific PCR primers and FullVelocity SYBR Green QPCR master mix (Stratagene, La Jolla, CA) using MyiQ real-time PCR detection system (BioRad, Hercules, CA). Each cycle threshold (CT) value was determined by iQ5 optical system software (BioRad), and normalized by the β-actin expression level. The primer sequences were designed by Primer Express software (Applied Biosystems, Foster City, CA), and their potential crossreactivity with other sequences were prescreened by In Silico PCR (University of California, Santa Cruz). The full sequences of the primers are posted in Supporting Information (Table S3).

### Western analysis

Total protein was extracted with RIPA buffer (20 mM Tris-HCl, pH 7.5, 150 mM NaCl, 1 mM Na_2_EDTA, 1 mM EGTA, 1% NP-40, 1% sodium deoxycholate, 2.5 mM sodium pyrophosphate, 1 mM β-glycerophosphate, 1 mM Na_3_VO_4_, and protease inhibitors). Protein concentrations were determined by BCA Protein Assay kit (Pierce, Rockford, IL). 50 µg of protein was separated by 10% SDS/PAGE and transferred onto a PVDF membrane (BioRad, Hercules, CA). The membrane was blocked in Odyssey blocking buffer (LI-COR Biotechnology, Lincoln, NA), and subsequently incubated with primary antibodies against Rho A (Santa Cruz Biotechnology, Santa Cruz, CA), E-cadherin (BD Biosciences), Rock I (BD Biosciences), Rock I (Millipore Corporation), Rock II (BD Biosciences), Stat3, phosphorylated Stat3 (tyr705), Akt, phospho-Akt, ERK1/2, phospho-specific ERK1/2 (Thr202/204) (Cell Signaling Technology, Beverly, MA), or β-actin (Sigma, St. Louis, MO) at 4°C overnight followed by incubation with peroxidase-conjugated goat anti-mouse IgG or goat anti-rabbit IgG (Jackson ImmunoResearch, Inc., West Grove, PA), and developed with ECL reagent (GE Healthcare, Piscataway, NJ).

### Rho kinase assay

The kinase activity of Rock was evaluated by using Rho-kinase assay kit (MBL International, Woburn, MA). The cell lysates extracted from ES cells treated with or without Y27632 at 10 µM for 24 hrs were incubated with kinase reaction buffer in the presence of ATP at 10 µM, and subsequently added to the substrate (recombinant myosin-binding subunit of myosin phosphatase, MBS, C terminus: 654–880)-coated multiwell plate. The phosphorylated substrate was recognized by horseradish peroxidase-conjugated phospho-MBS threonine-696-specific antibody that catalyzes the chromogenic substrate (tetra-methylbenzidine), and subjected to the colorimetric measurement by Spectramax microplate reader (Molecular Devices, Sunnyvale, CA).

### Embryoid body (EB) formation

hES cells were harvested by using dispase or trypsin-EDTA, plated on non-tissue culture treated dishes (approximately 10^7^ cells/ 10 cm dish) in the absence of Y27632, and grown in non-conditioned medium for 7 days. For the detection of differentiated derivatives, hES cell-derived EBs were plated on gelatin-coated 12-well plates to allow them to adhere, grown in DMEM (Invitrogen) containing 10%FBS (HyClone, Logan, UT) or Primary Neuron Growth Media (Lonza) to induce further differentiation for 14 days, and fixed in 4% paraformaldehyde followed by immunocytochemical analyses.

### Teratoma formation

All animal-related protocols were approved by Institutional Animal Care and Use Committee. hES cells (approximately 2×10^6^ cells) grown under animal-derived extracellular matrices-free culture conditions for multiple passages with occasional freezing and thawing were subcutaneously injected into severe combined immunodeficient (SCID)/ beige mice (Charles River Laboratories, Wilmington, MA). After four to eight weeks, the developed teratomas were excised, fixed in 4% paraformaldehyde or 10% formalin, and subjected to histological section preparations.

### Chimera experiment

mES cells (E14 or CJ7) were plated on gelatin or poly-D-lysine-coated plates (BD Biosciences) at a low density (500 cells/ cm^2^) in ESGRO Complete Clonal Grade medium in the presence or absence of Y27632 at 10 µM for 6 to 7 passages over 3 weeks with occasional freezing and thawing. Approximately ten to fifteen cells were microinjected into each blastocyst harvested from C57/Bl6 background mice. The injected blastocysts were introduced into foster mice. Chimerism of live offspring was confirmed by evaluation of their mixed coat color. Germ-line transmitters were determined by mating male chimeras with wild type C57/Bl6 female mice.

### Statistical analysis

Biological replicates (5 replicates per condition) were subjected to statistical analysis by using analysis of variance (ANOVA) or paired t-test. The statistical significance was shown as the probability value such as **p<0.001. Data points are shown as mean±standard deviation (SD).

## Supporting Information

Table S1 Evaluation of germ-line transmitters in chimeras derived from the Rock inhibitor-treated ES cells.
(0.03 MB DOC)

Table S2 siRNA sequences.
(0.11 MB DOC)

Table S3 Real-time QPCR primer sequences.
(0.06 MB DOC)

Figure S1 Evaluation of cell-cell contact states by ultrastructural and fluorescent microscopic analyses. (A) Representative images of undifferentiated mES cells grown under the control condition or in the presence of C3 exoenzyme for 24 hrs evaluated by transmission electron microscope (TEM). Specialized junctional complexes are indicated by arrows. (B) Immunofluorescent images of ES cells treated with or without C3 exoenxyme. E-cadherin and Oct3/4 localizations were determined by using specific primary antibodies in combination with secondary antibodies conjugated with Alexa Fluophore 488 (green) and 555 (red), respectively. Arrows indicate the intensified linear accumulation of E-cadherin in C3-treated cells. (C) A summary of the total number of cells that have undifferentiated stem cell features (high nuclear/cytoplasmic [N/C] ratio) and the number of undifferentiated cells that possess specialized junctional complexes as determined by TEM. Similar results were obtained from three independent experiments. Scale bars, 15 µm.
(4.60 MB TIF)

Figure S2 siRNA-mediated gene silencing in mES cells. To determine the transfection efficiency of siRNA in mES cells, cells were transfected with green fluorophore (FAM)-tagged siRNA at 40 nM with use of Lipofectamine 2000, and 24 hrs later, cells were evaluated on fluorescent inverted microscope. Virtually almost all cells incorporated fluorophore-tagged siRNA. Inset denotes the phase contrast image. To evaluate the level of knockdown of endogenous genes by siRNA treatment, mES cells were transfected with each siRNA targeting specific gene at 40 nM or at 25 nM when two distinct siRNAs were combined. 24 hrs later, cells were harvested, and subjected to Western or QPCR analysis. For Western analysis, β-actin was used as a loading control. Although the effect of Rock I siRNA appeared to be isoform-specific, Rock II siRNA affected both isoforms in a similar manner. Hence, the effect of Rock siRNA on the cell-cell contact was evaluated only when both Rock I and Rock II were simultaneously depleted by the cotransfection of Rock I and Rock II siRNAs. For the QPCR analysis, the data were normalized to the expression level of β-actin. The endogenous mRNA level of myosin IIC in mES cells was two orders below that of other myosins (data not shown). Although myosin IIC-specific siRNA treatment further reduced the expression level down to 40% that of the control, no substantial effects on cell-cell contact was observed (data not shown). Scale bars, 25 µm.
(1.63 MB TIF)

Figure S3 Morphological dynamics of mES cells triggered by Y27632 treatment or removal. (A) Morphology of E14 mES cells grown in the presence or absence (control) of Y27632 at 10 µM for 48 hrs. (B) mES cells were grown in the presence of Y27632 at 10 µM for 3 days. Subsequently, the compound was removed from the medium, and cells were photographed at 24 hrs and 48 hrs after the compound removal. Note that cells fully restore the normal cell-cell contact state at 48 hrs after Y27632 removal. Scale bars, 25 µm.
(4.47 MB TIF)

Figure S4 Activation states of the signal transduction pathways in mES cells. mES cells were grown in the presence or absence of Y27632 at 10 µM for several passages, and subjected to Western analysis using antibodies specific for major effector molecules downstream of each pluripotency-related signaling pathway.
(0.96 MB TIF)

Figure S5 hES cells grown in the absence of animal-derived extracellular matrices. (A) hES cells expressing GFP under the control of the Oct3/4 promoter were plated on non-tissue culture-treated dishes with conditioned medium supplemented with Y27632 at 10 µM. The GFP expression and morphology were sequentially captured by fluorescence microscopy. Note that GFP expression representing Oct3/4 promoter activity is equally inherited to two daughter cells. (B) Additional representative images showing the expression of IGF1 receptor (IGF1R) and Oct3/4 in hES cells passaged for an extended period on the PDL plate with mTeSR medium containing Y27632 at 10 µM (PDL). Note that no Oct3/4-negative fibroblastic cells physically surround the Oct3/4-positive undifferentiated cells grown on the PDL plate. The images on the bottom (low power view of the same area shown in Figure 6B) depict cells grown under the regular feeder-free condition (Matrigel). Scale bars, 25 µm.
(5.99 MB TIF)
