# Supplementary material for: The Rho-Rock-Myosin Signaling Axis Determines Cell-Cell Integrity of Self-Renewing Pluripotent Stem Cells
Source: PLoS One. 2008 Aug 20;3(8):e3001. doi: 10.1371/journal.pone.0003001 (PMC2500174; doi:10.1371/journal.pone.0003001)
Supplement: Table S1 — Evaluation of germ-line transmitters in chimeras derived from the Rock inhibitor-treated ES cells. (0.03 MB DOC) [file pone.0003001.s001.doc]

**Supplementary Table S1.**

Chimeras

Injection Males Females Germ-line transmitters

12/14/07 4 3 2 (2 of 4 males tested)

01/18/08 2 0 1 (1 of 2 males tested)
