# Supplementary material for: The Rho-Rock-Myosin Signaling Axis Determines Cell-Cell Integrity of Self-Renewing Pluripotent Stem Cells
Source: PLoS One. 2008 Aug 20;3(8):e3001. doi: 10.1371/journal.pone.0003001 (PMC2500174; doi:10.1371/journal.pone.0003001)
Supplement: Table S2 — siRNA sequences. (0.11 MB DOC) [file pone.0003001.s002.doc]

**Supplementary Table S2**

**siRNA sequences**

| **Target gene** | **Pool duplex** | **Primer** | **Sequence** |
| --- | --- | --- | --- |
| Rock I | 1 | Sense | GCAAAGAGAUUGUUAGAAU |
|  |  |  |  |
|  | 2 | Sense | AGACACAGCUGUAAGAUUA |
|  |  |  |  |
|  | 3 | Sense | UGUCGAAGAUGCCAUGUUA |
|  |  |  |  |
|  | 4 | Sense | GACCUUCAAGCACGAAUUA |
|  |  |  |  |
| Rock II | 1 | Sense | GAGAUUACCUUACGGAAAAUU |
|  |  | Antisense | 5'-PUUUUCCGUAAGGUAAUCUCUU |
|  |  |  |  |
|  | 2 | Sense | GGACAUGAGUUUAUUCCUAUU |
|  |  | Antisense | 5'-PUAGGAAUAAACUCAUGUCCUU |
|  |  |  |  |
|  | 3 | Sense | GCAAUGAAGCUUCUUAGUAUU |
|  |  | Antisense | 5'-PUACUAAGAAGCUUCAUUGCUU |
|  |  |  |  |
|  | 4 | Sense | CACAACAGAUGAUCAAAUAUU |
|  |  | Antisense | 5'-PUAUUUGAUCAUCUGUUGUGUU |
|  |  |  |  |
| Dia 1 | 1 | Sense | GUACAGCUGUGCGUGUUUG |
|  |  |  |  |
|  | 2 | Sense | GAAGUUGUCUGUAGAGGAA |
|  |  |  |  |
|  | 3 | Sense | GGAACAGUAUAACAAACUA |
|  |  |  |  |
|  | 4 | Sense | GAAACCAGCAUGAGAUUAU |
|  |  |  |  |
| Dia 2 | 1 | Sense | GAUGACCGAUCUUUGAUUUUU |
|  |  | Antisense | 5’-PAAAUCAAAGAUCGGUCAUCUU |
|  |  |  |  |
| Myosin IIA | 1 | Sense | GAGCGAGCCUCCAGGAAUAUU |
|  |  | Antisense | 5'-PUAUUCCUGGAGGCUCGCUCUU |
|  |  |  |  |
|  | 2 | Sense | GCACCAAGCUCAAGCAGAUUU |
|  |  | Antisense | 5'-PAUCUGCUUGAGCUUGGUGCUU |
|  |  |  |  |
|  | 3 | Sense | GAACCGAACUGGCCGACAAUU |
|  |  | Antisense | 5'-PUUGUCGGCCAGUUCGGUUCUU |
|  |  |  |  |
|  | 4 | Sense | GAAGGUGGCUGCCUACGAUUU |
|  |  | Antisense | 5'-PAUCGUAGGCAGCCACCUUCUU |
|  |  |  |  |
| Myosin IIB | 1 | Sense | GGACUUAUCUAUACUUACUUU |
|  |  | Antisense | 5'-PAGUAAGUAUAGAUAAGUCCUU |
|  |  |  |  |
|  | 2 | Sense | GAGCGUACAUUUCAUAUCUUU |
|  |  | Antisense | 5'-PAGAUAUGAAAUGUACGCUCUU |
|  |  |  |  |
|  | 3 | Sense | UGAGGCAGCUAGUAUUAAAUU |
|  |  | Antisense | 5'-PUUUAAUACUAGCUGCCUCAUU |
|  |  |  |  |
|  | 4 | Sense | GUAUUAAGUUUGCGAAGGAUU |
|  |  | Antisense | 5'-PUCCUUCGCAAACUUAAUACUU |
|  |  |  |  |
| Myosin IIC | 1 | Sense | CUGAAGAAAGACCGCAAUAUU |
|  |  | Antisense | 5'-PUAUUGCGCUCUUUCUUCAGUU |
|  |  |  |  |
|  | 2 | Sense | UCAAGGACCAUUACCGAAAUU |
|  |  | Antisense | 5'-PUUUCGGUAAUGGUCCUUGAUU |
|  |  |  |  |
|  | 3 | Sense | ACGCAGAGGUAGAGCGCGAUU |
|  |  | Antisense | 5'-PUCGCGCUCUACCUCUGCGUUU |
|  |  |  |  |
|  | 4 | Sense | AGGCGGAACUUGAGAGCGUUU |
|  |  | Antisense | 5'-PACGCUCUCAAGUUCCGCCUUU |
|  |  |  |  |
| MYPT1 | 1 | Sense | GAACGAGACUUGCGUAUGUUU |
|  |  | Antisense | 5'-PACAUACGCAAGUCUCGUUCUU |
|  |  |  |  |
|  | 2 | Sense | AAGAAUAGUUCGAUCAAUGUU |
|  |  | Antisense | 5'-PCAUUGAUCGAACUAUUCUUUU |
|  |  |  |  |
|  | 3 | Sense | CGACAUCAAUUACGCCAAUUU |
|  |  | Antisense | 5'-PAUUGGCGUAAUUGAUGUCGUU |
|  |  |  |  |
|  | 4 | Sense | UCGGCAAGGUGUUGAUAUAUU |
|  |  | Antisense | 5'-PUAUAUCAACACCUUGCCGAUU |
