# Supplementary material for: The Rho-Rock-Myosin Signaling Axis Determines Cell-Cell Integrity of Self-Renewing Pluripotent Stem Cells
Source: PLoS One. 2008 Aug 20;3(8):e3001. doi: 10.1371/journal.pone.0003001 (PMC2500174; doi:10.1371/journal.pone.0003001)
Supplement: Table S3 — Real-time QPCR primer sequences. (0.06 MB DOC) [file pone.0003001.s003.doc]

**Supplementary Table S3**

**Real-time QPCR primer sequences**

| mouse_beta-actin | Forward | 5’- CGAGGCCCAGAGCAAGAG-3’ |
| --- | --- | --- |
|  | Reverse | 5’- CGTCCCAGTTGGTAACAATGC-3’ |
| mouse_Rock I | Forward | 5’- ATTCATTCCTACCCTCTACCACTTTC-3’ |
|  | Reverse | 5’- GCTTAAAGACATGCCACAAAGGT-3’ |
| mouse_Rock II | Forward | 5’- GCGATGCTGAGCCTGATGAT-3’ |
|  | Reverse | 5’-GCACAGGCAATGACAACCAT-3’ |
| mouse_Dia 1 | Forward | 5’- GGATGCACAGGAACAGTATAACAAA-3’ |
|  | Reverse | 5’- AAGACGAAGTAGTCACCTAGCTCCTT-3’ |
| mouse_Dia 2 | Forward | 5’- CAGTCAGGTGCAGCATTCAGA-3’ |
|  | Reverse | 5’-GGGTCTTACCTGGATTTCTTGGA-3’ |
| mouse_Myosin IIA | Forward | 5’- TGGCAAGCAAGCGTGTGT-3’ |
|  | Reverse | 5’- GCCGATGCGGTACAGGTT-3’ |
| mouse_Myosin IIB | Forward | 5’- TCCTCACGCCCAGGATCA-3’ |
|  | Reverse | 5’- GCCAATGCTTCCACTGCAA-3’ |
| mouse_Myosin IIC | Forward | 5’- GCAATGCCAAGACGGTGAA-3’ |
|  | Reverse | 5’- GATGTAGCCAGCAATATCAAAGTTGA-3’ |
| mouse_MYPT1 | Forward | 5’- GACTCCCCCGGGTTCCT-3’ |
|  | Reverse | 5’- CCTCAGCCCACAAACGATTT-3’ |
| mouse_Pou5f1 | Forward | 5’- TGGCGTGGAGACTTTGCA-3’ |
|  | Reverse | 5’- GAGGTTCCCTCTGAGTTGCTTTC-3’ |
| mouse_Nanog | Forward | 5’- TGTGCACTCAAGGACAGGTTTC-3’ |
|  | Reverse | 5’- CAGGTTCAGAATGGAGGAGAGTTC-3’ |
| mouse_FGF5 | Forward | 5’- CAACACGTCTCCACCCACTTC-3’ |
|  | Reverse | 5’- TTTCTGGAACAGTGACGGTGAA-3’ |
| mouse_Snail1 | Forward | 5’- CCACTGCAACCGTGCTTTT-3’ |
|  | Reverse | 5’- CACATCCGAGTGGGTTTGG-3’ |
| mouse_N-Cadherin | Forward | 5’- GGGTCTGTTCCAGAGGGATCA-3’ |
|  | Reverse | 5’- GGATCATCCGCATCAATGG-3’ |
| human_Oct3 | Forward | 5’- GAAGCCTTTCCCCCTGTCTCT-3’ |
|  | Reverse | 5’- AAGGGCAGGCACCTCAGTT-3’ |
| human_Nanog | Forward | 5’- AATGAAATCTAAGAGGTGGCAGAAA-3’ |
|  | Reverse | 5’- TTCTGCGTCACACCATTGCT-3’ |
| human_FGF5 | Forward | 5’- GCAGCCCCCGGGTTAA-3’ |
|  | Reverse | 5’- GCTCCGACTGCTTGAATCTTG-3’ |
| human_Slug | Forward | 5’- CCATTCCACGCCCAGCTA-3’ |
|  | Reverse | 5’- TCACTCGCCCCAAAGATGAG-3’ |
| human_GATA6 | Forward | 5’- GGATTGTCCTGTGCCAACTGT-3’ |
|  | Reverse | 5’- GGTTCACCCTCGGCGTTT-3’ |
