# Supplementary figures and images for: The Rho-Rock-Myosin Signaling Axis Determines Cell-Cell Integrity of Self-Renewing Pluripotent Stem Cells
Source: PLoS One. 2008 Aug 20;3(8):e3001. doi: 10.1371/journal.pone.0003001 (PMC2500174; doi:10.1371/journal.pone.0003001)

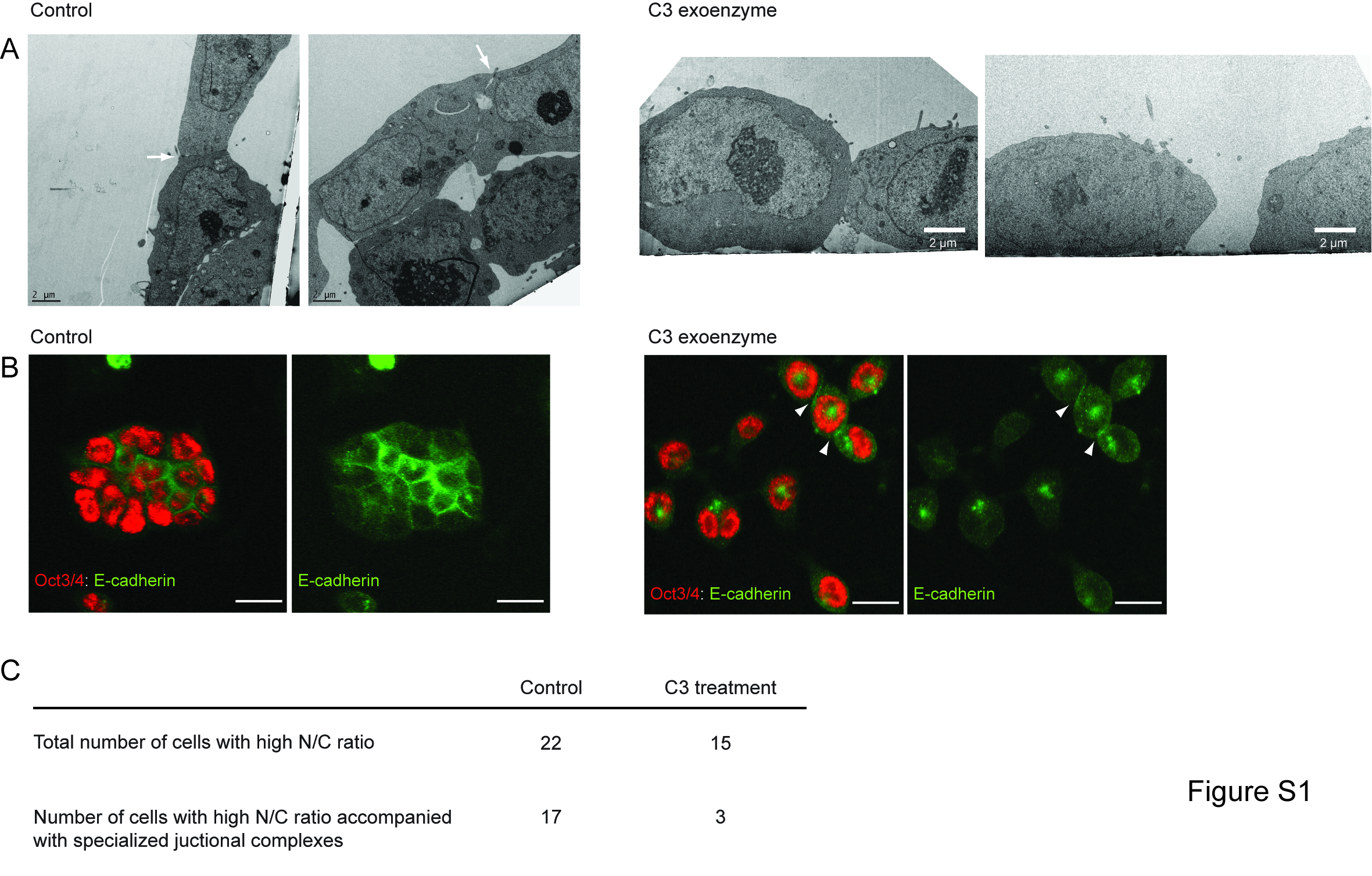

Supplement: Figure S1 — Evaluation of cell-cell contact states by ultrastructural and fluorescent microscopic analyses. (A) Representative images of undifferentiated mES cells grown under the control condition or in the presence of C3 exoenzyme for 24 hrs evaluated by transmission electron microscope (TEM). Specialized junctional complexes are indicated by arrows. (B) Immunofluorescent images of ES cells treated with or without C3 exoenxyme. E-cadherin and Oct3/4 localizations were determined by using specific primary antibodies in combination with secondary antibodies conjugated with Alexa Fluophore 488 (green) and 555 (red), respectively. Arrows indicate the intensified linear accumulation of E-cadherin in C3-treated cells. (C) A summary of the total number of cells that have undifferentiated stem cell features (high nuclear/cytoplasmic [N/C] ratio) and the number of undifferentiated cells that possess specialized junctional complexes as determined by TEM. Similar results were obtained from three independent experiments. Scale bars, 15 µm. (4.60 MB TIF) [file pone.0003001.s004.tif]

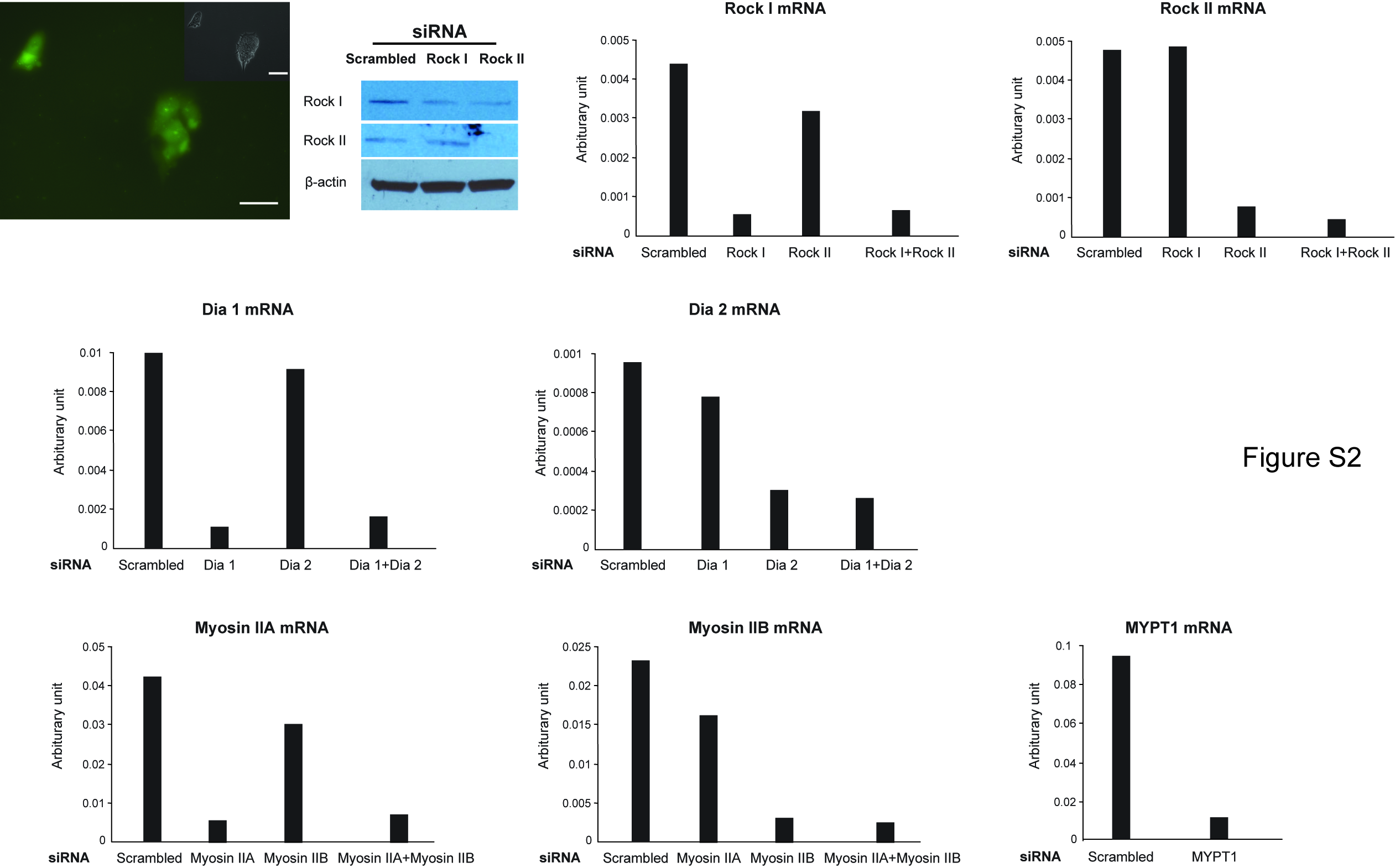

Supplement: Figure S2 — siRNA-mediated gene silencing in mES cells. To determine the transfection efficiency of siRNA in mES cells, cells were transfected with green fluorophore (FAM)-tagged siRNA at 40 nM with use of Lipofectamine 2000, and 24 hrs later, cells were evaluated on fluorescent inverted microscope. Virtually almost all cells incorporated fluorophore-tagged siRNA. Inset denotes the phase contrast image. To evaluate the level of knockdown of endogenous genes by siRNA treatment, mES cells were transfected with each siRNA targeting specific gene at 40 nM or at 25 nM when two distinct siRNAs were combined. 24 hrs later, cells were harvested, and subjected to Western or QPCR analysis. For Western analysis, β-actin was used as a loading control. Although the effect of Rock I siRNA appeared to be isoform-specific, Rock II siRNA affected both isoforms in a similar manner. Hence, the effect of Rock siRNA on the cell-cell contact was evaluated only when both Rock I and Rock II were simultaneously depleted by the cotransfection of Rock I and Rock II siRNAs. For the QPCR analysis, the data were normalized to the expression level of β-actin. The endogenous mRNA level of myosin IIC in mES cells was two orders below that of other myosins (data not shown). Although myosin IIC-specific siRNA treatment further reduced the expression level down to 40% that of the control, no substantial effects on cell-cell contact was observed (data not shown). Scale bars, 25 µm. (1.63 MB TIF) [file pone.0003001.s005.tif]

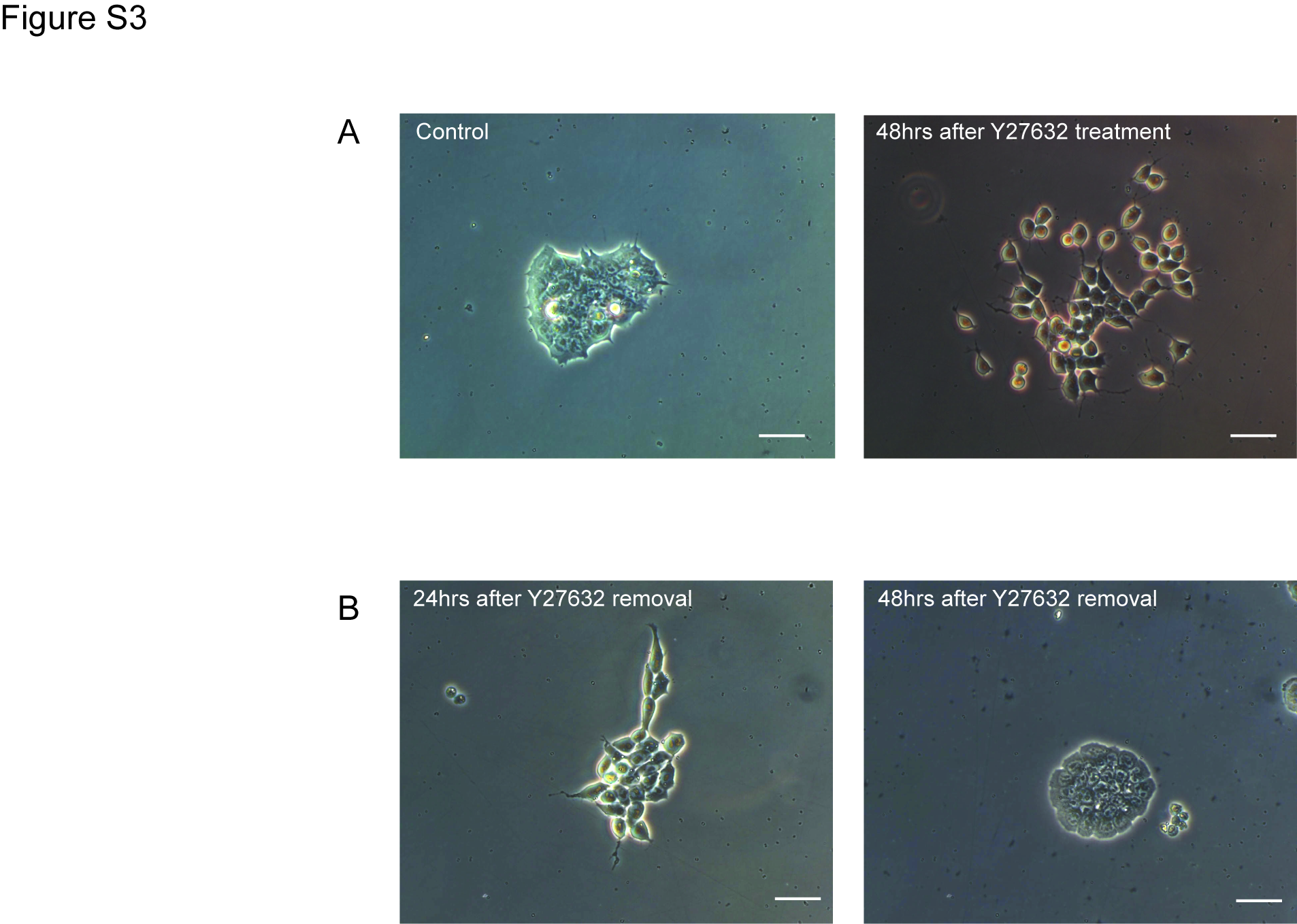

Supplement: Figure S3 — Morphological dynamics of mES cells triggered by Y27632 treatment or removal. (A) Morphology of E14 mES cells grown in the presence or absence (control) of Y27632 at 10 µM for 48 hrs. (B) mES cells were grown in the presence of Y27632 at 10 µM for 3 days. Subsequently, the compound was removed from the medium, and cells were photographed at 24 hrs and 48 hrs after the compound removal. Note that cells fully restore the normal cell-cell contact state at 48 hrs after Y27632 removal. Scale bars, 25 µm. (4.47 MB TIF) [file pone.0003001.s006.tif]

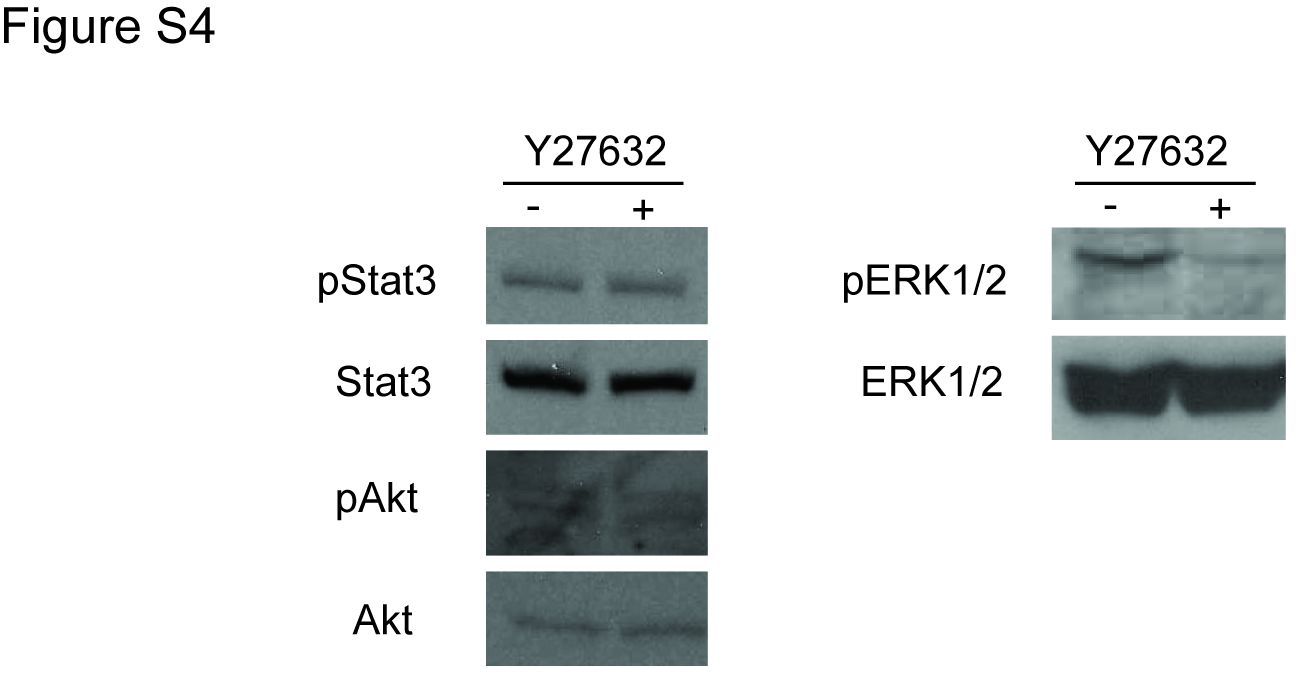

Supplement: Figure S4 — Activation states of the signal transduction pathways in mES cells. mES cells were grown in the presence or absence of Y27632 at 10 µM for several passages, and subjected to Western analysis using antibodies specific for major effector molecules downstream of each pluripotency-related signaling pathway. (0.96 MB TIF) [file pone.0003001.s007.tif]

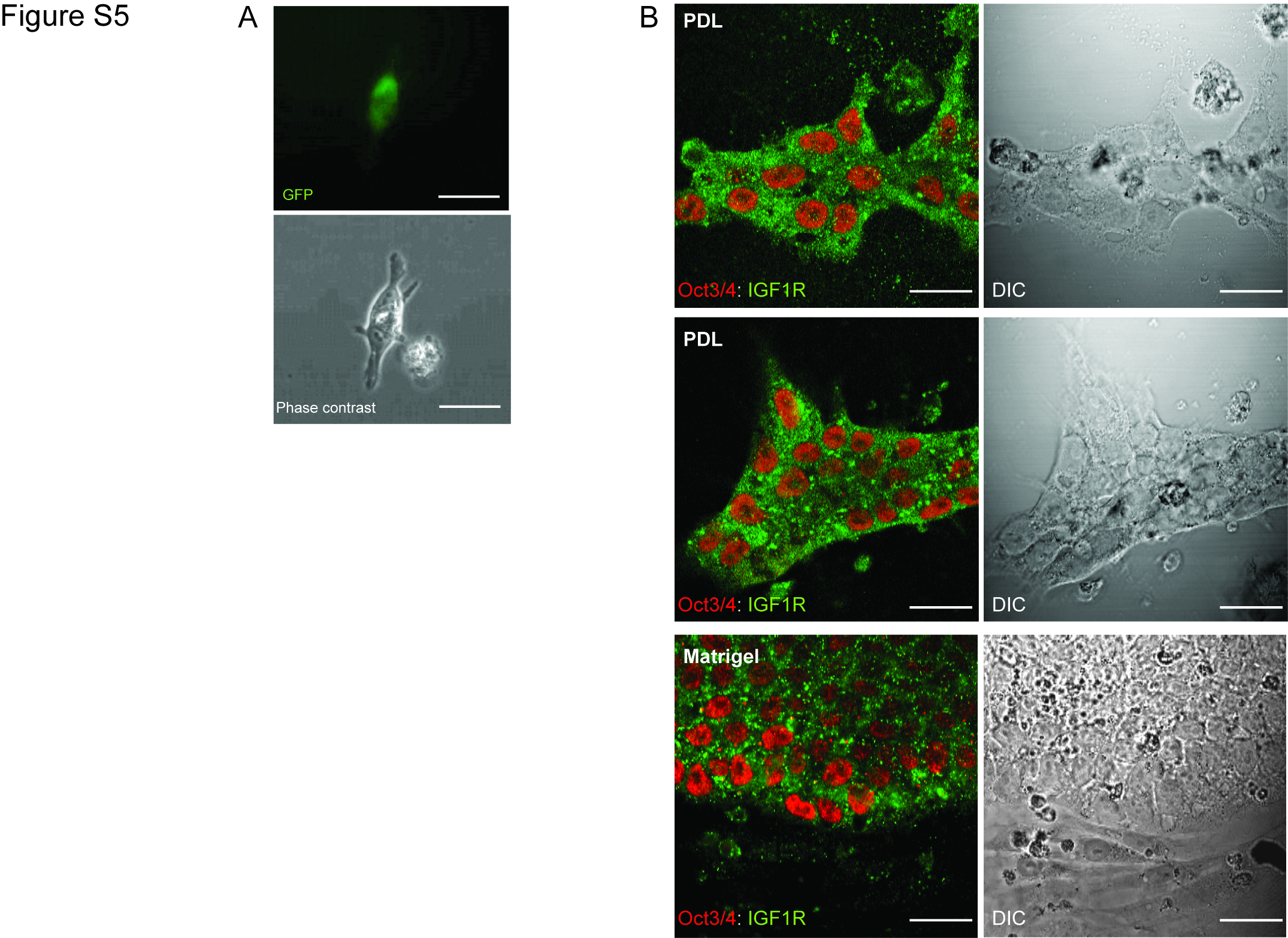

Supplement: Figure S5 — hES cells grown in the absence of animal-derived extracellular matrices. (A) hES cells expressing GFP under the control of the Oct3/4 promoter were plated on non-tissue culture-treated dishes with conditioned medium supplemented with Y27632 at 10 µM. The GFP expression and morphology were sequentially captured by fluorescence microscopy. Note that GFP expression representing Oct3/4 promoter activity is equally inherited to two daughter cells. (B) Additional representative images showing the expression of IGF1 receptor (IGF1R) and Oct3/4 in hES cells passaged for an extended period on the PDL plate with mTeSR medium containing Y27632 at 10 µM (PDL). Note that no Oct3/4-negative fibroblastic cells physically surround the Oct3/4-positive undifferentiated cells grown on the PDL plate. The images on the bottom (low power view of the same area shown in Figure 6B) depict cells grown under the regular feeder-free condition (Matrigel). Scale bars, 25 µm. (5.99 MB TIF) [file pone.0003001.s008.tif]
